# Frontiers of Oncology: Biobanking Resources for the 21^st ^Century

**DOI:** 10.1186/2043-9113-1-13

**Published:** 2011-05-08

**Authors:** Jill Barnholtz-Sloan, Mark R Chance

**Affiliations:** 1 Case Comprehensive Cancer Center and Center for Proteomics and Bioinformatics, Case Western Reserve University School of Medicine, Cleveland, Ohio, USA

## 

The progress of translational research and enhancing the prospects for personalized medicine require coordinated efforts across a wide range of disciplines as well as public-private partnerships of many kinds. Advances in genomics and proteomics technologies are providing more accurate data on more targets and more types of targets than ever before. These include whole genome sequence information; identification and quantification of thousands of specific protein targets, including details of a myriad of protein modifications; epigenetic modifications; and new frontiers that include micro RNAs and protein and nucleic acid interactome complexities. Our technological advances will continue unabated, as it is unlikely that we have plumbed the depths of biological complexity governing development and disease.

Clinical databases and the digitization of clinical information have improved significantly along with the advances in -omics technologies. Significant challenges in defining and enforcing ontologies and encouraging meta-data capture represent significant hurdles. The Cancer Biomedical Informatics Grid (CaBIG) and other tissue database projects have developed common dictionaries of terms for cancer staging and defining diagnostic subclasses of cancer; such efforts are critical to being able to query across databases. In this issue Surati and colleagues at the University of Chicago outlined their success in developing a Thoracic Oncology Database that serves as a repository for well-annotated cancer specimens combined with clinical, genomic, and proteomic data obtained from specific tumor tissue studies. Their goal was to make the database not just a repository, but also a dynamic tool to drive data mining and exploratory analysis for clinical and translational research for thoracic oncology. In the article, the investigators used non-small cell lung cancer samples from the database combined with specific proteomic analyses of these samples to examine functional relevance of protein over- and under-expression. Clinical data for 1323 patients with non-small cell lung cancer was captured and proteomic studies were performed on tissue samples from 105 patients. Initial biomarker studies identified receptor tyrosine kinase family members that were over-expressed in tumor tissues. Since clinical data and research data are present in a single database, investigators were able to powerfully address research questions or plan studies that minimize duplication, maximize the potential for valuable results and encourage additions to the database by other investigators at the University of Chicago and at other institutions. In fact the stated goals of the study, as outlined in the accompanying paper were to: (1) create a platform to house clinical, genomic, and proteomic data from patients with thoracic malignancies; (2) tailor the platform to meet the needs of clinical and basic science researchers; and (3) utilize the platform in support of meaningful statistical analysis to correlate laboratory and clinical information.

It is generally understood that well-annotated clinical specimens are fundamental to advancing translational medicine and clinical oncology care. For oncology, in particular, the union of clinical and biologic data is at the highest level of evolution. Standardization of biospecimen collection and consent processes, processing and annotation of biospecimens and prioritization of specimen use for translational research is a top priority for the National Cancer Institute (NCI). To standardize and disseminate best practices the NCI has developed the Office of Biorepositories and Biospecimen Research (OBBR) http://biospecimens.cancer.gov/default.asp. Several other ongoing initiatives with NCI and the National Human Genome Research Institute (NHGRI) are currently implementing standards as set forth by OBBR; these initiatives include the Cancer Genome Atlas Project (TCGA) http://tcga.cancer.gov/, the Cancer Genome Anatomy Project (CGAP) http://cgap.nci.nih.gov/), the International Cancer Genome Consortium (ICGC) http://www.icgc.org/, the Repository of Molecular Brain Neoplasia Data (REMBRANDT) https://caintegrator.nci.nih.gov/rembrandt/, the International HapMap Project http://hapmap.ncbi.nlm.nih.gov/ and the 1000 Genomes Project http://www.1000genomes.org/. In addition, other NCI programs focused on therapeutic clinical trials for cancer patients have also begun to implement OBBR's standards for their biobanking initiatives; e.g., the NCI's Cancer Therapy Evaluation Program (CTEP, http://ctep.cancer.gov), the Early Detection Research Network (EDRN, http://edrn.nci.nih.gov), and the Cooperative Oncology Groups http://www.cancer.gov/cancertopics/factsheet/. All of these resources serve to connect discovery from biospecimens to the broader cancer research community and building intelligent links between individual institution and NCI and cooperative group clinical trials is critical since this entire network provides the largest collection of similarly treated individuals, many of whom do not have tissue acquisitions.

The Cancer Genome Atlas (TCGA) Project serves to foster groundbreaking medical research using standardized procedures for multi-site patient consent, biospecimen collection, processing, banking and clinical annotation (which also includes active patient follow-up). The overarching goal of TCGA is to improve our ability to diagnose, treat and prevent cancer via full molecular characterization of more than 20 cancer types. RNA and DNA from the same set of tumor and matched normal samples are analyzed by multiple characterization centers for copy number variation, chromosomal segment aberrations, loss of heterozygosity, epigenetic alterations, gene and miRNA expression changes as well as mutation by large-scale sequencing. TCGA has an established, integrated network of clinical sites, core resources, specialized genome characterization and genome sequencing centers and incorporates powerful bioinformatics and data analysis centers. The Cancer Proteomics Centers http://proteomics.cancer.gov/ will now be responsible for adding proteomics data to this resource. As technologies are evolving rapidly, the concept of "full" molecular characterization is a moving target. However, TCGA has evolved and will continue to evolve along with the technologies, with more and more scientific papers and discoveries that promise to ultimately lead to advances in cancer prevention and treatment.

Going forward, the question as to how academic projects, such as that outlined by Surati and colleagues the University of Chicago, may be linked and leveraged with public projects is not yet answered. Agreements of standards and sharing of data and resources, especially those that have been funded by public dollars, are critical to progress. A role for cancer advocacy organizations can be envisioned in this framework as they could play advocate and/or provide seed funding for biobank projects of specific interest to their donors, and this seed funding could come with important strings attached, namely that projects must be designed and delivered for the benefit of the patients and the public. If the advocacy groups can help build the resources, academic institutions may be best suited to properly exploiting these with testable hypotheses. However, this will require transparent and fair access policies for samples and data as well as cost recovery that maintains these resources.

The recent identification of genetic mutations linked to pathogenesis and clinical behavior in breast cancer, colon cancer and glioblastoma and many other cancers have advanced our knowledge in terms of clinical diagnosis and treatment tremendously. In fact, we can only expect more powerful markers leading to robust patient stratification as the quality of biobanks and their annotations and the technologies used to acquire these data improve. Some of the important next steps in oncology management will require proteomics data to be available along with the existing comprehensive genomic data as technologies to analyze gene and protein expression data in the context of gene and protein networks are providing specific predictions for functional protein level dysregulation. In addition, since proteomic expression information is only modestly correlated with gene expression data (e.g. r2 ~ 0.5-0.6) more careful examination where correlation is lacking may reveal the involvement of novel pre- and posttranscriptional regulators such as miRNAs and ubiquitination, respectively

In conclusion, momentum in building biobanking resources with well-annotated clinical specimens is growing, which can only further facilitate and accelerate translational research discoveries. The NIH is clearly concerned for the future of translation; the development and maintenance of the Centers for Translational Science will be critical to this future as will the execution of plans for the National Center for Advancing Translational Sciences. Individually, we need to monitor strategic plans at our own institutions to see that biospecimen resources do not become museums but dynamic and evolving research resources. This will require adequately vetted business plans with careful analysis of scientific return on investment. Developing regional networks through our academic medical centers in collaboration with primary care health care networks is the next logical step. Ultimately national and international level collaborations might become the norm. In the meantime, the importance of properly annotated biospecimen collection needs to be highlighted among the research community, patients and the general public. In the future, patient oriented research could become more efficient and more effective, leading to better treatment decisions, including when not to treat, and improved clinical outcomes.

